# Calcium Prevents Tumorigenesis in a Mouse Model of Colorectal Cancer

**DOI:** 10.1371/journal.pone.0022566

**Published:** 2011-08-17

**Authors:** Ji-Lin Wang, Yan-Wei Lin, Hui-Min Chen, Xuan Kong, Hua Xiong, Nan Shen, Jie Hong, Jing-Yuan Fang

**Affiliations:** 1 Division of Gastroenterology, Shanghai Jiao-Tong University School of Medicine, Renji Hospital, Shanghai Institute of Digestive Disease, Shanghai, China; 2 Division of Rheumatology, Shanghai Jiao-Tong University School of Medicine, Renji Hospital, Shanghai, China; Enzo Life Sciences, Inc., United States of America

## Abstract

**Background and Aim:**

Calcium has been proposed as a mediator of the chemoprevention of colorectal cancer (CRC), but the comprehensive mechanism underlying this preventive effect is not yet clear. Hence, we conducted this study to evaluate the possible roles and mechanisms of calcium-mediated prevention of CRC induced by 1,2-dimethylhydrazine (DMH) in mice.

**Methods:**

For gene expression analysis, 6 non-tumor colorectal tissues of mice from the DMH + Calcium group and 3 samples each from the DMH and control groups were hybridized on a 4×44 K Agilent whole genome oligo microarray, and selected genes were validated by real-time polymerase chain reaction (PCR). Functional analysis of the microarray data was performed using KEGG and Gene Ontology (GO) analyses. Hub genes were identified using Pathway Studio software.

**Results:**

The tumor incidence rates in the DMH and DMH + Calcium groups were 90% and 40%, respectively. Microarray gene expression analysis showed that *S100a9*, *Defa20*, *Mmp10*, *Mmp7*, *Ptgs2*, and *Ang2* were among the most downregulated genes, whereas *Per3*, *Tef*, *Rnf152*, and *Prdx6* were significantly upregulated in the DMH + Calcium group compared with the DMH group. Functional analysis showed that the Wnt, cell cycle, and arachidonic acid pathways were significantly downregulated in the DMH + Calcium group, and that the GO terms related to cell differentiation, cell cycle, proliferation, cell death, adhesion, and cell migration were significantly affected. *Forkhead box M1* (*FoxM1*) and *nuclear factor kappa-B* (*NF-κB*) were considered as potent hub genes.

**Conclusion:**

In the DMH-induced CRC mouse model, comprehensive mechanisms were involved with complex gene expression alterations encompassing many altered pathways and GO terms. However, how calcium regulates these events remains to be studied.

## Introduction

The current incidence of colorectal cancer (CRC) is high, and its prognosis remains poor [1]; hence, it is important to devise strategies to prevent the development of CRC. Many agents are used to prevent CRC [2]; however, there is no consistency in the effects across agents [3], [4], [5], and some agents may lead to severe side effects [6], [7]. Thus, an alternative approach is necessary for the prevention of CRC.

A recent systemic review revealed that calcium may be more attractive in the prevention of CRC compared with aspirin and screening [8]. However, the effectiveness of calcium is still uncertain. Pooled findings of 10 cohort studies revealed a reverse relationship between calcium intake and CRC risk [9], but this relationship was not confirmed by a randomized controlled clinical trial [10]. However, due to the limitations of the randomized control trial (RCT), e.g., low dose of calcium, short length of follow-up, and influence of estrogen intake [11], the conclusion from the RCT was not convincing. Nonetheless, the effectiveness of calcium on the prevention of CRC remains controversial [12].

Similarly, the mechanism of calcium-mediated prevention of CRC is not yet clear. In previous studies, calcium was thought to reduce the risk of CRC by binding to toxic secondary bile acids and forming insoluble soaps in the lumen of the colon [13], or by reducing proliferation, stimulating differentiation, and inducing apoptosis in the colonic mucosa [14], [15]. A recent study suggested that calcium could abrogate hyperplasia in the distal colon by inhibiting a calcium channel receptor, i.e., transient receptor potential channel, subfamily V, member 6 (TRPV6) [16]. These findings indicate that calcium could reduce the risk of CRC through a variety of mechanisms, but the specific and comprehensive mechanism is yet not clear. Therefore, we conducted this study to evaluate the possible roles and mechanisms of calcium-mediated prevention of CRC induced by 1,2-dimethylhydrazine (DMH) in mice. We believe that our findings will contribute to the research on the clinical application of calcium.

## Materials and Methods

### Ethics Statement

Our study was approved by the Animal Care and Use Committee of the Shanghai Jiao-Tong University School of Medicine Renji Hospital, Shanghai, China. All animal procedures were performed according to the guidelines developed by the China Council on Animal Care and the protocol approved by the Shanghai Jiao-Tong University School of Medicine, Renji Hospital, Shanghai, China.

### Chemicals

DMH was obtained from Sigma Chemical Co. (St. Louis, MO,USA). Normal and high calcium feed for mice were obtained from Shanghai SLAC Laboratory Animal Company (Shanghai, China). The main components of the normal and high calcium feed were the same: protein, 22.1%; fat, 5.28%; ash, 5.2%; fiber, 4.12%; nitrogen-free extract, 52%; phosphorus, 0.92%; and Vitamin D3 6818.4 IU/kg. The concentration of calcium (in the form of calcium carbonate) in the normal and high calcium feed was 1.24% and 3.0%, respectively. The high calcium feed was adjusted by adding calcium carbonate to obtain a final calcium content of 3%. Because we think that the controversial effectiveness of high calcium diet on the prevention of CRC may be partially due to the small dose of calcium in previous studies, so we decided to use higher calcium content to test the exact preventive effect of CRC and the mice's tolerance.

### Experimental animals

In total, 80 female Slac: ICR mice [weight, 18–20 g; grade, specific pathogen-free (SPF)] were purchased from the Chinese Academy of Sciences (Shanghai, China). They were maintained at room temperature (22°C) with a relative humidity of 60% and 12-hour light/dark cycles; they were supplied a standard laboratory diet and drinking water. The mice were randomly divided into 4 groups (20 mice in each group): Control group, DMH group, DMH+Calcium group, and Calcium group. The mice of the Control group were provided normal feed and received subcutaneous injections of normal saline; those of the DMH group were provided normal feed and received subcutaneous injection of DMH at a dose of 20 mg/kg once weekly for 20 weeks; those of the DMH+Calcium group were provided high-calcium feed and received subcutaneous injection of DMH at a dose of 20 mg/kg once weekly for 20 weeks; those of the Calcium group were provided high-calcium feed and received subcutaneous injections of normal saline. The mice were killed at the end of 24 weeks, and the incidence of CRC in each group was examined. This method has been described in detail in our previous study [17]. In brief, longitudinal incisions were made in colorectal tissues to observe the number of gross tumors. After then, the gross tumors were removed separately and the full-thickness colorectal tissues were cutted in half by the longitudinal axis. Some of the freshly obtained tumor samples together with their half corresponding non-tumor colorectal tissues were frozen immediately in liquid nitrogen. The remaining samples were fixed in formalin solution and embedded in paraffin blocks for pathological analysis and immunohistochemistry.

### Histological analysis

For histological analysis, 4-µm-thick sections of formalin-fixed, paraffin-embedded colon tumors were prepared. After hematoxylin and eosin staining, the sections of each tumor were examined under a light microscope (Olympus, Japan). The results were confirmed by the pathologist Chen XY.

### RNA extraction, labeling, hybridization, and analysis

Total RNAs from 12 non-tumor colorectal tissues [3 from the Control group, 3 from the DMH group, 6 from the DMH + Calcium group (among the 6 from the DMH + Calcium group, 3 were from mice with tumors and 3 were from mice without tumors)] were harvested using TRIzol (Invitrogen) according to manufacturer's instruction. The RNA content was measured using a Nanodrop ND-1000 spectrophotometer, and denaturing gel electrophoresis was conducted. Next, the samples were amplified, labeled using the Agilent Quick Amp labeling kit, and hybridized using the Agilent whole genome oligo microarray (Agilent Technologies, Palo Alto, CA) by using Agilent SureHyb hybridization chambers. After hybridization and washing, the processed slides were scanned with the Agilent DNA microarray scanner using the settings recommended by Agilent Technologies.

The resulting text files extracted from the Agilent Feature Extraction Software (version 10.5.1.1) were imported into the Agilent GeneSpring GX software (version 11.0) for further analysis. Background intensity has been cut off before normalization. The microarray data sets were normalized in GeneSpring GX using the Agilent FE one-color scenario (mainly median normalization). Differentially expressed genes were identified by determining the fold-change (FC) and p values of the *t*-test. Genes with an FC of ≥1.5 and a p value of ≤0.05 between 2 groups were identified as differentially expressed genes. Functional analysis of the differentially expressed genes was performed using Gene Ontology (GO) (http://www.geneontology.gov/) [18] and the KEGG PATHWAY Database (http://www.genome.jp/kegg/pathway.html). Hub genes were identified using Pathway Studio (Ariadne Genomics) [19].

### Real-time polymerase chain reaction

Reverse transcription was performed using oligo (dT) primers and superscript II reverse transcriptase (Takara). Quantification of gene expression was performed using a real-time polymerase chain reaction (PCR) kit (Takara). The expression level of 18 s rRNA was used as an internal control. The expression of the following genes was analyzed: *S100a9*, *Defa20*, *Mmp10*, *Ptgs2*, *Per3*, *Tef*, *Rnf152*, and *Prdx6*. The primers are listed in Table 1.

**Table 1:** Primer sequence.

Gene name	Forward sequence	Reverse sequence	Product length
S100a9	5′-AACATCTGTGACTCTTTAGCCTTG-3′	5′-ACTGTGCTTCCACCATTTGTCT-3′	169
Mmp10	5′-CCCAGCTAACTTCCACCTTTC-3′	5′-AGCAGGATCACATTTGTCTGG-3′	142
Defa20	5′-AGGCTGTGTCTGTCTCCTTTG-3′	5′-TGAGCAGGTCCCATAAACTTG-3′	128
Ptgs2	5′-GAGTGGGGTCATGAGCAACTA-3′	5′-CTGGAACTGCTGGTTGAAAAG-3′	153
Per3	5′-CACCTCTTCGAGTGAGTCCAG-3′	5′-CCAGTATCCGTGGTGCTTTTA-3′	246
Tef	5′-TCTTCCTCTACTGCCATCTTTCA-3′	5′-AAGTTCACATCGACCTCCACAC-3′	122
Rnf152	5′-AGTCATTGCCATACCACACACTT-3′	5′-GAGTGTACGCTCCTTAGAGATGG-3′	106
Prdx6	5′-AAGTTAGACACACAGCCACAA-3′	5′-ACTTGGCACCGTAGTTTTGTTT-3′	120
18S rRNA	5′-CGGACAGGATTGACAGATTGATAGC-3′	5′-TGCCAGAGTCTCGTTCGTTATCG-3′	150

### Immunohistochemistry

Four-micrometer-thick sections of formalin-fixed, paraffin-embedded non-tumor colon tissues were deparaffinized and rehydrated. The microwave repair method was used for antigen retrieval. Endogenous peroxidase activity was blocked by incubation of the sections with 0.3% hydrogen peroxide for 10 min. Non-specific antigen was blocked by incubation of the sections with sheep serum for 30 min. Slides were incubated overnight at 4°C in a humidified chamber with rabbit monoclonal anti-β-catenin (Cell Signaling Technology, #9562) at a dilution of 1∶200. Anti-rabbit IgG was used as secondary antibody (30 min incubation). Diaminobenzidine was used as chromogen and the sections were counterstained with hematoxylin. Samples incubated with PBS instead of primary antibody were used as negative controls.

### Statistical analysis

The results of the animal experiments and real-time PCR were analyzed using SAS 9.2 software (SAS Institute Inc. USA). Data are presented as means ± SD. Student's *t*-test was used to compare values between 2 independent groups.

## Results

### Animal experiments

**Figure 1:**
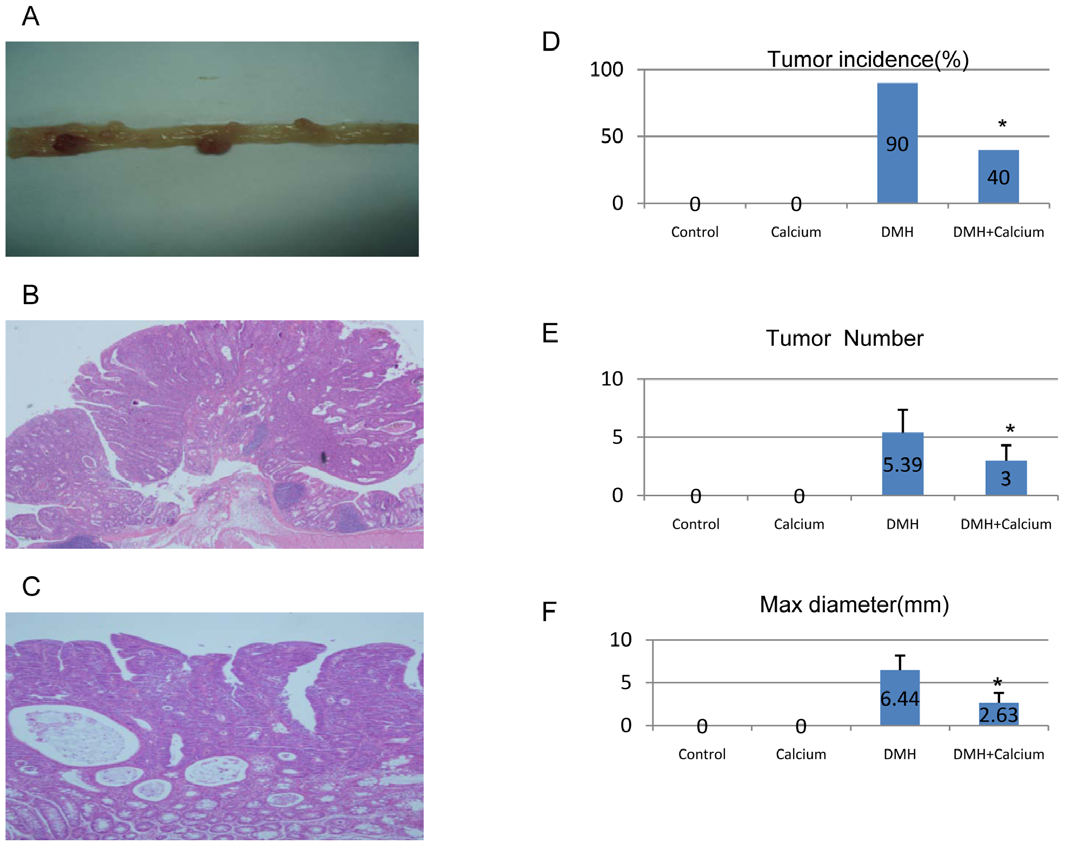
Main results of the animal experiment. *A.* Observation of tumors in the large bowel of mice after sacrifice at 24 weeks. *B* (×200) and *C* (×400). Most of the tumors were confirmed as adenocarcinomas by pathological examination. *D*. Tumor incidence among the 4 groups. *E*. Tumor number/mice among the 4 groups. *F*. Maximum tumor diameter among the 4 groups (Control: Control group, received normal feed and injection of normal sodium for 20 weeks; DMH: DMH group, received normal feed and injection of DMH for 20 weeks; DMH+Calcium group: received high-calcium feed and injection of DMH for 20 weeks; Calcium group: received high-calcium feed and injection of normal sodium for 20 weeks). * *P* value<0.05 between DMH+Calcium group and DMH group.

The main results of the animal experiments are shown in Figure 1. We successfully induced CRC in mice using DMH (Figure 1A). Most of the cancers were identified as adenocarcinomas by histological analysis (Figure 1B, 1C). In the DMH and DMH+Calcium groups, the tumor incidence was 90% and 40% (Figure 1D), the mean number of tumors per mouse was 5.39±1.97 and 3.0±1.31 (Figure 1E), and mean maximum diameter of the tumors was 6.44±1.72 and 2.63±1.19 (Figure 1F), respectively. No tumor was found in the Control and Calcium groups. Growth and development of the mice in the Calcium group were not significantly affected, and the pathological examination of their kidneys, hearts, lungs, livers, and spleens revealed no obvious abnormalities (data not shown).

### Gene expression profile by microarray analysis

All 12 colonic tissues cleared the quality control step and were analyzed as described in the Methods section. The microarray analysis was conducted between the Control group (3 samples), DMH group (3 samples), and DMH + Calcium group (6 samples). We also compared the gene expression levels between the samples with or without tumors among the DMH + Calcium group.

**Figure 2:**
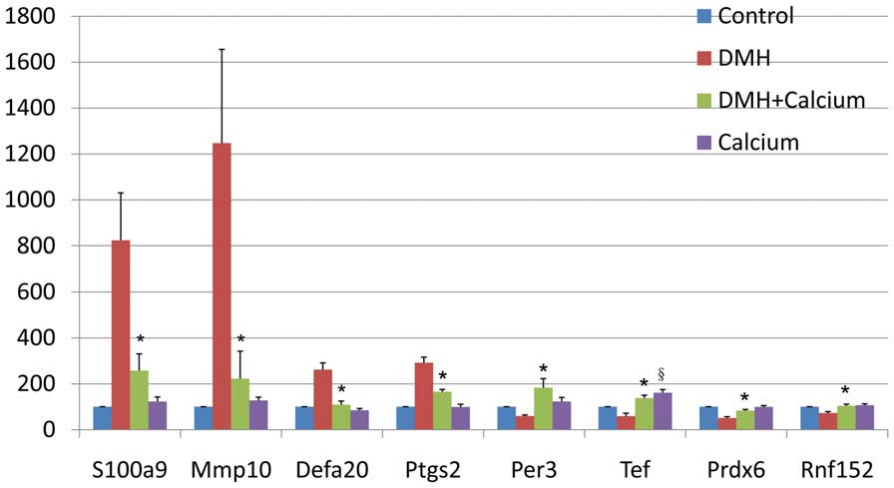
Validation of differentially expressed genes by real-time polymerase chain reaction. We used 18 s rRNA as an internal control. Relative mRNA expression was calculated according to the 2^−ΔΔT^ method. Data are the mean of 10 samples ± SD. Control, gene expression level in the normal colon tissue of mice in the Control group; DMH, gene expression level in the non-tumor colon tissue of mice in the DMH group; DMH+Calcium, gene expression level in the non-tumor colon tissue of mice in the DMH+Calcium group; Calcium, gene expression in the non-tumor colon tissue of mice in the Calcium group. * *P* value<0.05 between DMH+Calcium group and DMH group; § *P* value<0.05 between Calcium group and Control group.

Hierarchical clustering analysis of the 12 array expression data showed a homogenous expression profile among the samples of each group (Figure S1). By setting the filter for the FC to ±1.5 and the p value at ≤0.05, we found that the expression of 12395 genes was significantly altered in the DMH group compared to those in the Control group (see Table S1), and that of 1508 genes was significantly altered in the DMH + Calcium group compared to those in the DMH group (see Table S2). Compared with Table S1 and Table S2, we found that the expression of 549 genes whose changes due to DMH treatment could be reversed by dietary calcium (see Table S3). In Table 2, the 30 most differentially expressed genes are shown, most of which are closely related to tumorigenesis, such as *S100a9* (S100 calcium-binding protein A9 [calgranulin B]), *Defa20* (defensin alpha 20), *Mmp10* (matrix metalloproteinase 10), *Ang2* (angiogenin, ribonuclease A family, member 2), *Per3* (period homolog 3), *Tef* (thyrotroph embryonic factor), and *Ptgs2* (prostaglandin-endoperoxide synthase 2). For the 8 selected genes, i.e., *S100a9*, *Defa20*, *Mmp10*, *Ptgs2*, *Per3*, *Tef*, *Rnf152*, and *Prdx6*, the results obtained from the microarray analysis were confirmed by real-time PCR (Figure 2). We selected these genes for PCR confirmation because they are closely related to tumorigenesis and worth further research.

**Table 2:** List of the most differentially expressed genes whose changes due to DMH treatment could be reversed by diatery calcium.

Accession number	Gene symbol	Gene Description	Fold change	P value
NM_007847	Defa-rs2	defensin, alpha, related sequence 2	−9.88	1.93E-04
NM_009114	S100a9	S100 calcium binding protein A9	−9.58	4.25E-05
NM_019471	Mmp10	matrix metallopeptidase 10	−9.47	7.67E-07
NM_008607	Mmp13	matrix metallopeptidase 13	−9.10	1.48E-04
NM_183268	Defa20	defensin, alpha, 20	−8.16	1.20E-04
NM_010810	Mmp7	matrix metallopeptidase 7	−7.07	0.00411071
EU817850	Gm5222	monoclonal antibody 1D6 immunoglobulin light chain variable region	−6.98	1.99E-05
NM_008764	Tnfrsf11b	tumor necrosis factor receptor superfamily, member 11b	−5.98	0.01292794
NM_011915	Wif1	Wnt inhibitory factor 1	−5.79	0.00447852
NM_001174099	Krt36	keratin 36	−5.79	2.75E-04
NM_009263	Spp1	secreted phosphoprotein 1	−5.40	1.31E-04
NM_175391	Apol7c	apolipoprotein L 7c	−4.93	5.03E-07
NM_007969	Expi	extracellular proteinase inhibitor	−4.78	3.00E-02
NM_008256	Hmgcs2	3-hydroxy-3-methylglutaryl-Coenzyme A synthase 2	−4.62	9.86E-06
NM_010743	Il1rl1	interleukin 1 receptor-like 1	−4.38	1.28E-05
NM_026414	Asprv1	aspartic peptidase, retroviral-like 1	−4.38	2.82E-05
NM_007449	Ang2	angiogenin, ribonuclease A family, member 2	−4.34	0.04452419
NM_053080	Aldh1a3	aldehyde dehydrogenase family 1, subfamily A3	−4.11	0.01939012
NM_007440	Alox12	arachidonate 12-lipoxygenase	−4.03	0.02319294
NM_001142959	Bcl2l15	BCLl2-like 15,transcript variant 1	−3.95	5.27E-04
NM_016958	Krt14	keratin 14	−3.81	0.00713059
NM_007753	Cpa3	carboxypeptidase A3	−3.74	3.03E-05
NM_008939	Prss12	protease, serine, 12 neurotrypsin	−3.65	0.04354441
NM_011280	Trim10	tripartite motif-containing 10	−3.58	3.46E-06
NM_001082531	Pla2g2a	phospholipase A2, group IIA	−3.50	8.61E-04
NM_021285	Myl1	myosin, light polypeptide 1	−3.31	9.72E-04
NM_011067	Per3	period homolog 3	3.16	2.08E-05
NM_153484	Tef	thyrotroph embryonic factor	2.98	1.26E-05
NM_011198	Ptgs2	prostaglandin-endoperoxide synthase 2	−2.87	1.53E-04
AK081980	Prdx6	16 days embryo head cDNA, product:peroxiredoxin 5	2.87	0.01674954
NM_178779	Rnf152	ring finger protein 152	2.63	0.0028878

Fold changs and P values are the results comparing DMH+Calcium group and DMH group.

**Figure 3:**
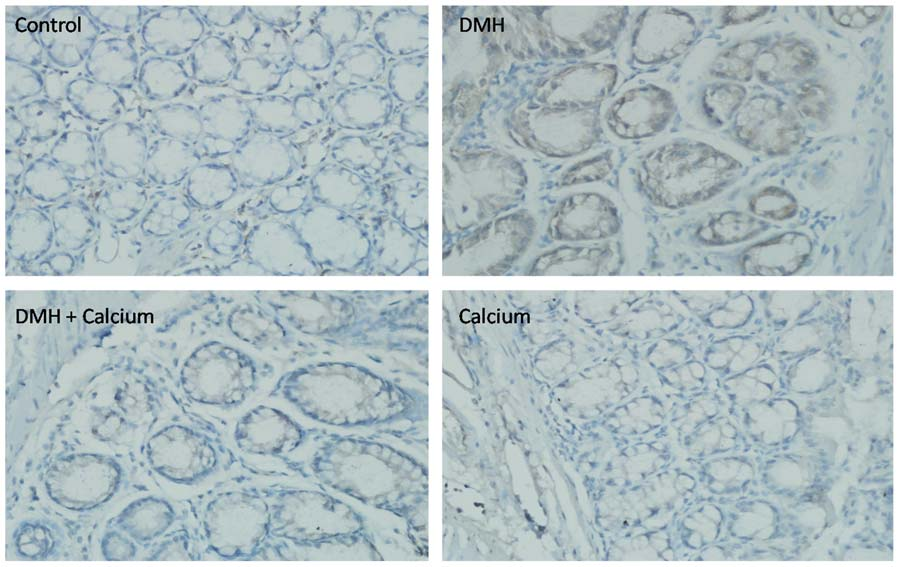
Expression of β-catenin in the non-tumor colorectal mucosa in the 4 groups determined by immunohistochemistry. Control, Control group; DMH, DMH group; DMH+Calcium, DMH+Calcium group; Calcium, Calcium group.

To determine whether specific biological pathways or functional gene groups were differentially affected by the high calcium diet, we analyzed our microarray dataset (on the basis of the results shown in Table S3) using the GO and KEGG software. The detailed results are shown in Table S4 and Table S5. By setting the p value at ≤0.05, we found significant downregulation of 39 signaling pathways, including some tumor-related pathways such as the cell cycle pathway, Wnt signaling pathway, vascular endothelial growth factor (VEGF) signaling pathway, and transforming growth factor (TGF-β) signaling pathway. The most enriched pathways are shown in Table 3. We used immunohistochemistry to detect the protein expression and distribution of β-catenin, the core molecule in the Wnt pathway, and found that its expression was significantly higher in the non-tumor colonic mucosa from the DMH group than in that from the Control group, however, its expression in the DMH + Calcium group was markedly reduced compared with the DMH group (Figure 3). Altogether, 703 GO terms, including tumor-related GO terms such as cell differentiation, cell proliferation, apoptosis, angiogenesis, cell adhesion, cell cycle, and cell division, were significantly changed.

**Table 3:** The most enrichment pathways by KEGG.

Pathway ID	Pathway name	Selection Count	Count	Enrichment
**Down-regulated pathways**				
mmu04310	Wnt signaling pathway	11	148	5.123285
mmu05140	Leishmaniasis	6	67	3.468583
mmu04110	Cell cycle	8	127	3.383628
mmu04370	VEGF signaling pathway	5	76	2.378304
mmu04010	MAPK signaling pathway	10	272	2.299372
mmu04114	Oocyte meiosis	6	114	2.277315
mmu04664	Fc epsilon RI signaling pathway	5	81	2.260629
mmu04350	TGF-beta signaling pathway	5	84	2.194259
mmu05222	Small cell lung cancer	5	84	2.194259
mmu04640	Hematopoietic cell lineage	5	85	2.172789
mmu00590	Arachidonic acid metabolism	5	86	2.151633
mmu04012	ErbB signaling pathway	5	88	2.110233
mmu00650	Butanoate metabolism	3	30	2.084872
mmu05216	Thyroid cancer	3	30	2.084872
**Up-regulated pathways**				
mmu00982	Drug metabolism - cytochrome P450	8	85	7.972006
mmu04710	Circadian rhythm - mammal	4	22	5.391566
mmu00591	Linoleic acid metabolism	3	45	2.827674
mmu00910	Nitrogen metabolism	2	23	2.231527
mmu00980	Metabolism of xenobiotics by cytochrome P450	3	75	2.197296
mmu00830	Retinol metabolism	3	78	2.150205
mmu03320	PPAR signaling pathway	3	81	2.105092

“SelectionCounts” stands for the Count of the DE genes' entities directly associated with the listed PathwayID;

“Count” stands for the count of the chosen background population genes' entities associated with the listed PathwayID;

“Enrichment_Score” stands for the Enrichment Score value of the PathwayID, it equals “−log10(Pvalue)”.

**Figure 4:**
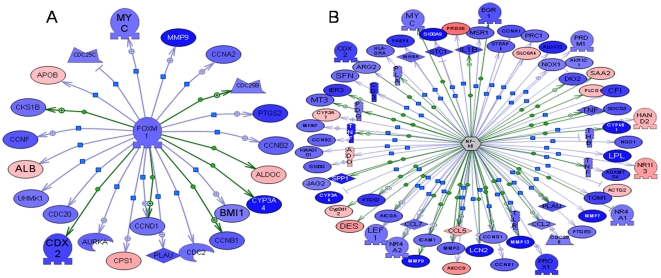
Potential hub genes derived from Pathway studio software. The differentially expressed genes regulated by the 2 hub genes (A,*FoxM1*;B, *NF-κB*) are displayed in form of a network diagram. Green or red coloring, sequences that are downregulated or upregulated in the DMH+Calcium group compared with the DMH group, respectively. The intensity of the color is proportional to the extent of change.

We also used Pathway Studio software to filter hub genes that may play key roles in the calcium-mediated prevention of CRC (Table 4). Of those, 2 genes were selected to construct gene networks (Figure 4). On the basis of the networks, we could clearly determine the core roles of the hub genes.

**Table 4:** Partial list of the hub genes.

Name	Total No.of targeted genes	No.of overlap genes	Percent overlap	Gene set seed	P value
Expression Targets of Jun/Fos	542	68	12%	Jun/Fos	1.55E-08
Expression Targets of PKA	369	52	14%	PKA	2.03E-08
Expression Targets of EGF	504	64	12%	EGF	2.71E-08
Expression Targets of FOXM1	108	24	22%	FOXM1	4.04E-08
Expression Targets of TP53	538	65	12%	TP53	1.41E-07
Expression Targets of MYOCD	36	13	35%	MYOCD	1.66E-07
Expression Targets of NF-kB	742	76	10%	NF-kB	8.28E-06
Expression Targets of TNF	900	87	9%	TNF	1.69E-05

In addition, we further compared the gene expression levels among the mice from the DMH + Calcium group with/without tumors and found that the gene expression levels of most of the above selected genes in mice from the DMH + Calcium group with tumors was between those from the DMH + Calcium group without tumors and those from the DMH group (see Table S6 and Figure S1). We also found that the expression of some calcium transporters was different among the mice from the DMH + Calcium group with/without tumors. For example, the expression of Trpv6 and Trpv3 was significantly upregulated in mice from the DMH + Calcium group with tumors compared to those without tumors; however, the expression of plasmalemmal Ca^2+^-ATPase (PMCA) was significantly downregulated.

## Discussion

In this study, we used the DMH-induced CRC mouse model that shows phenotypic and genotypic features similar to those observed in human sporadic CRCs and, thus, can be applied to study the prevention of CRC [20]. In this study, we found that the incidence of CRC decreased significantly in mice fed a high calcium diet, indicating a clear role of calcium in the prevention of CRC. Our results are consistent with many other studies. Pence [21] have found that rats on the high calcium diet have a decreased CRC incidence compared to the low calcium diet (86% vs. 53%), Salas [22] also observed a significant diminution in the number of tumors and a decrease in the CRC incidence in the group fed high calcium diet (97% vs. 64%). However, some studies have observed different outcomes. Sitrin [23] found that neither calcium supplementation alone nor supplemental calcium conjunction with vitamin D deficiency altered the incidence of CRC induced by DMH, Mølck [24] even found an increased incidence in the rats fed high calcium diet. We think that the differences in the animal strain, animal age, rearing environment, basis diet, DMH doses and duration of injection, calcium concentration, calcium intervention time may be accounted for the differences in the outcome of various studies using high calcium. The confirmed preventive effectiveness of CRC with high calcium diet could not yet be contained from those studies. We think it is may be due to the small doses of calcium in those studies. So we used higher calcium content(3%) in the feed to test the preventive effect of CRC and the mice' tolerance. In fact, this high calcium feed showed good preventive effect of CRC in our experiment and no obvious adverse effects was found in mice. In addition, no tumor was found in the Calcium groups. Growth and development of the mice in the Calcium group were not significantly affected, and the pathological examination of their kidneys, hearts, lungs, livers, and spleens revealed no obvious abnormalities, the expression of most of the selected genes confirmed by real-time PCR in the Calcium group was equal to that in the Control group. These findings indicate that this high calcium diet is safe for the mice.

Next, we used microarray gene expression profile analysis to determine the mechanism of calcium-mediated prevention of CRC. To the best of our knowledge, this is the first investigation to use microarray technology for studying the role of high calcium diet in the prevention of CRC.

When the FC was set to ≥1.5, the changes in 549 genes that were the result of DMH treatment could be reversed with dietary calcium. *S100a9* is the most downregulated gene; its product is a cytoplasmic Ca^2+^-binding protein. Although extracellular S100a9 could induce apoptosis by binding to a yet unknown receptor and cause dimerization of Bax and Bak and their translocation to the mitochondria leading to mitochondrial damage [25], its expression in epithelial cells could indeed induce cell proliferation by activating the phosphoinositide 3-kinase (PI3K)–Akt–NF-κB survival pathway, which is associated with tumorigenesis [26]. In addition, S100a9 is an important proinflammatory cytokine involved in innate immunity, and its expression was found to be elevated in numerous pathological conditions associated with inflammation [27]. Many other inflammatory cytokines, such as Defa, were also significantly downregulated [members of the defensin family were downregulated at different levels: *Defa-rs2* (FC = −9.88), *Defa20* (FC = −8.16)]. Tumor necrosis factor (*Tnf*) and its receptors were also downregulated at different levels: *Tnf* (FC = −1.87), *Tnfrsf11b* (FC = −5.98), *Tnfrsf19* (FC = −2.27). Several other studies performed using the DMH or azoxymethane (AOM)-induced CRC model also found high expression levels of S100a9 and Defa in tumor tissues [28], [29]. Inflammatory cytokines play important roles in the innate and adaptive immunity; however, those elevated cytokines are also involved in tumor initiation, promotion, and invasion [30], [31], [32], [33], and thus, play an important role in inflammation-associated carcinogenesis [34].

MMPs are a family of matrix metalloproteinases that can play important roles in the tumor microenvironment [35]. Many members of the MMP family were significantly downregulated in the DMH + Calcium group [*MMP10* (FC = −9.47), *MMP13* (FC = −9.1), *MMP7* (FC = −7.07), and *MMP11* (FC = −1.56)]. Other metalloproteinases were also downregulated, such as *Adam8* and *Adam10*, which were downregulated by 2.08 and 1.6 times, respectively. Recent studies have shown that many members of the MMP and ADAM family could play various roles in tumorigenesis. For example, *MMP7* could cleave Fas ligand, thereby lowering the impact of chemotherapy on the tumor by abrogating apoptosis [36]. *Adam10* may trigger the release of soluble epidermal growth factor (EGF), mediate the shedding of E-cadherin, translocating β-catenin to the nucleus, and thus, driving cell proliferation [37]. However, the most important role of the members of the MMP and ADAM family is promoting tumor invasion and metastases by degrading extracellular matrix [38], [39]. These findings indicate that a high calcium diet may play an important role in inhibiting invasion and metastases of CRC. Because *in vitro* studies showed that, in CRC cells, E-cadherin expression could be induced by increasing the extracellular Ca^2+^ concentration [40], calcium may be considered to suppress the epithelial-mesenchymal transition (EMT) to inhibit CRC metastases.

*Per3* was the most upregulated gene in our study, belonging to the period genes. Those genes are core elements of the transcriptional/translational feedback loops that generate the endogenous circadian rhythm, and *Per3* participates in timekeeping in the pituitary and lung [41]. Recent studies have suggested that circadian genes participate in the growth and development of various cancers, and the period genes have now been linked to DNA damage response pathways, inhibition of the growth of cancer cells, and increase in the apoptotic rate [42]. Oshima's study has shown that the expression of *Per3* in CRC tissues was significantly lower than that in the adjacent normal mucosa, suggesting that *Per3* may function as a tumor suppressor gene [43].

*Tef* was the second most upregulated gene in our study. It belongs to the PAR-domain basic leucine zipper (PAR bZip) transcription factors and is involved in detoxification and drug metabolism [44] and in the maintenance of cell shape in fibroblasts [45]. It also serves as a pro-apoptotic gene by promoting the expression of bcl-gs or bik [46], [47]. Many other genes were also upregulated, e.g., *Rnf152* and *Prdx6*. *Rnf152* is a novel RING finger protein and has been shown to have pro-apoptotic activity [48]. *Prdx6* is a member of the PRDX family, associated with functions such as cell proliferation, differentiation, and apoptosis, thus executing anti-cancer activity [49].

By using pathway analysis, we found that the Wnt pathway was significantly affected. Although the expression of the Wnt inhibitory factor *wif1* was significantly downregulated, that of many genes associated with the Wnt signaling pathway and their target genes was downregulated [i.e., *Tcf7*, *Myc*, *cyclin d1* (*Ccnd1*), and *Mmp7* at FC of −1.53, −1.61, −1.62, and −7.07, respectively]. The protein expression of β-catenin was significantly reduced in the DMH + Calcium group, suggesting that the Wnt pathway was downregulated in this group. This is consistent with previous findings [28], [50]. Interestingly, the cell cycle pathway was also significantly inhibited in the DMH + Calcium group, with many genes in this pathway being consistently downregulated, i.e., the cyclins *Ccnb1* and *Ccnd1* were downregulated by 1.79 and 1.62 times, respectively, and the expression of the cyclin-dependent kinase *Cdk1* was reduced by 1.66 times.

Among the metabolic pathways, the arachidonic acid pathways were significantly inhibited in the DMH + Calcium group. Many enzymes in this pathway showed reduced expression, e.g., Ptgs2, also known as cyclooxygenase-2 (COX-2), was reduced by 3 times. The arachidonic acid pathway is activated during inflammation, leading to the synthesis of prostaglandin and thromboxane, which play an important role in inflammatory response [51]. Furthermore, the key enzyme in this pathway, Ptgs2 (COX-2), plays an important role in the initiation of CRC. COX-2 overexpression was shown to be correlated with carcinogenesis in more than 80% of CRCs [52]. In animal models, COX-2 expression was found to be sufficient to induce tumorigenesis [53]. The mechanism may be related to the activation of inducible nitric oxide synthase (iNOS) and VEGF signaling to promote formation of new blood vessels [54]. On the basis of the finding that the expression of the inflammatory cytokines S100a9, Defa, TNF, and TNF receptors was also significantly downregulated in the DMH+Calcium group, we speculated that calcium may play an important role in reducing inflammation-related tumorigenesis.

Among the Pathway Studio-derived hub genes, which may play key roles in the calcium-mediated prevention of CRC, *FoxM1*, *NF-κB*, etc., were also involved. FoxM1 belongs to a family of evolutionarily conserved transcriptional regulators that were characterized by the presence of a DNA-binding domain known as the forkhead box domain. Higher expression of FoxM1 was noted in many types of human cancers [55]. FoxM1 may play important roles in cell proliferation and apoptosis [56]. The molecular mechanism underlying FoxM1 signaling-mediated induction of tumor growth has not been completely elucidated. However, multiple oncogenic pathways such as PI3K/Akt, NF-κB, extracellular signal-regulated kinase (ERK), mitogen-activated protein kinase (MAPK), VEGF, reactive oxygen species (ROS), c-myc, and hypoxia-inducible factor (HIF)-1 have been reported to interact with FoxM1 signaling; this suggests that the interaction between FoxM1 signaling and other signaling pathways may play important roles in tumorigenesis [57]. It should be noted that the expression of many genes regulated by NF-κB showed altered expression in the DMH + Calcium group, e.g., *TNF*, *PTGS2*, *MYC*, *CCND1*, *ETS-related gene 1* (*ERG1*), *S100A9*, and *lymphoid enhancer factor 1* (*LEF1*), suggesting that NF-κB may also serve as a hub gene in the calcium-mediated prevention of CRC. Although the expression of NF-κB did not change significantly at the transcriptional level, it may have changed at the translational or posttranslational level. The expression pattern of NF-κB in the DMH + Calcium group and its specific mechanism needs to be further elucidated.

We also found that the gene expression levels of most of the above selected genes in mice from the DMH+Calcium group with tumors was between those from the same group without tumors and those from the DMH group, thus confirmed the importance of those genes in the calcium's prevention of CRC. Interestingly, the expression of some calcium transporters, such as Trpv6, Trpv3 and PMCA, was different among the mice from the DMH + Calcium with/without tumors. One of the biological functions of these calcium transporters is to mediate transcellular Ca^2+^ movement across epithelial cells [58]. Therefore, the differential expression of these calcium transporters disrupted the distribution of intracellular and extracellular calcium, thereby activating the proliferation-related pathways [59]. Hence, we speculated that the differential expression of these calcium transporters may partially account for the different expression levels of the selected genes.

It is necessary to mention the limitations of this study. First, we used colorectal tissues instead of colorectal mucosa in our microarray study. There are many different types of cells in the colorectal tissues, so it is hard to determine the cellular origin of the identified genes in this study. However, more and more evidences have shown that the stroma and immune cells play important roles in the tumor initiation. In our studies, we have found that there are many immune cells infiltrated into the colorectal tissues by pathological examination and the expression of many inflammation cytokines is significantly upregulated in the DMH group compared with Control group. These cells can not only cause local inflammation, but also significantly promote tumorigenesis [34]. We also have found that high calcium diet could reduce the expression of many inflammation cytokines, suggesting that calcium may play an important roles in the prevention of inflammation-related tumorigenesis. Therefore we believe that using colon tissues instead of colon mucosa could reflect the overall and more realistic changes in the gene expression. To our knowledge, colon tissues are widely used in the study of gene expression associated with CRC [60], [61]. Second, since we did not use semi-purified diets in our animal experiment, our results may be biased. Third, the calcium content in our study was rather high. Although this may account for the drastic effectiveness in the prevention of CRC and no obvious adverse effect was found in the mice in our study, it is difficult to apply it for the prevention of sporadic CRC in humans, because exceedingly high calcium and vitamin D concentrations in the diet may pose a risk for developing hypercalcemia and kidney stones. However, a meta-analysis found that a calcium intake of more than 1000 mg/day will have a better preventive effect [62]. Another clinical trial found that a lower calcium intake may have no apparent preventive effect [10]. Therefore, further study is necessary to confirm the optimal calcium content in the prevention of CRC.

In conclusion, we confirmed the effectiveness of calcium to prevent CRC in the DMH-induced CRC mouse model. We also clarified the comprehensive mechanisms of calcium-mediated prevention of CRC. Our results showed that calcium exerted its protective effect mainly by downregulating the expression of oncogenes such as *S100a9*, *Mmp10*, *Adam8*, and *Ptgs2*, by inhibiting the activity of tumor-associated pathways such as the Wnt and cell cycle pathway, and by negatively regulating cell proliferation, cell division, cell invasion, and angiogenesis. In particular, calcium is speculated to play important roles in reducing inflammation-associated tumors and suppressing tumor invasion and metastases. However, the mechanism of the calcium-mediated regulation of these genes remains to be studied.

## Supporting Information

Table S1 **Complete list of differentially expressed genes in the DMH group compared with the Control group.**
(XLS)

Table S2 **Complete list of differentially expressed genes in the DMH + Calcium group compared with the DMH group.**
(XLS)

Table S3 **Complete list of genes whose changes due to DMH treatment could be reversed by dietary calcium.**
(XLS)

Table S4 **Complete list of the GO terms based on the genes whose changes due to DMH treatment could be reversed by dietary calcium.**
(XLS)

Table S5 **Complete list of pathways based on the genes whose changes due to DMH treatment could be reversed by dietary calcium.**
(XLS)

Table S6 **Complete list of differentially expressed genes in the DMH + Calcium group with/without tumors.**
(XLS)

Figure S1 **Hierarchical clustering of the 1.5-fold upregulated and downregulated genes whose changes due to the DMH treatment could be reversed by dietary calcium.** Control, Control group; DMH + Calcium, DMH + Calcium group; DMH, DMH group. CA-1, 2, 3, samples from the DMH + Calcium group without tumors; CA2-1, 2, 3, samples from the DMH + Calcium group with tumors.
(TIF)
