# Supplementary figures and images for: Calcium Prevents Tumorigenesis in a Mouse Model of Colorectal Cancer
Source: PLoS One. 2011 Aug 17;6(8):e22566. doi: 10.1371/journal.pone.0022566 (PMC3157344; doi:10.1371/journal.pone.0022566)

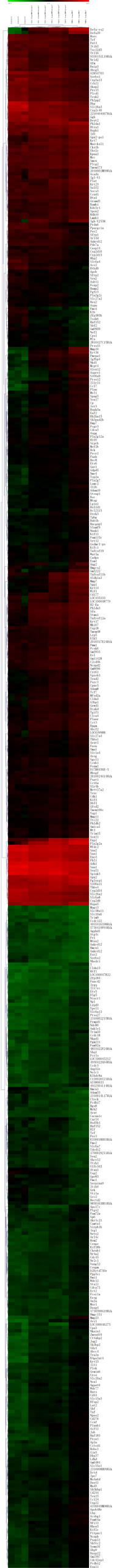

Supplement: Figure S1 — Hierarchical clustering of the 1.5-fold upregulated and downregulated genes whose changes due to the DMH treatment could be reversed by dietary calcium. Control, Control group; DMH + Calcium, DMH + Calcium group; DMH, DMH group. CA-1, 2, 3, samples from the DMH + Calcium group without tumors; CA2-1, 2, 3, samples from the DMH + Calcium group with tumors. (TIF) [file pone.0022566.s007.tif]
